# Bone health and fracture risk in diabetes: A multicenter study

**DOI:** 10.1007/s11657-026-01667-z

**Published:** 2026-02-16

**Authors:** Özen Öz Gül, Zeynep Cantürk, Sadettin Öztürk, Nilüfer Özdemir, Dilek Kılınç Candemir, Burak Özbaş, Filiz Mercantepe, Filiz Mercan Sarıdaş, Erhan Hocaoğlu, Onur Elbasan, Burcu Candemir, Berrin Çetinarslan, Ali Saklamaz, Şerife Ezgi Doğan, Elif Seray Korkmaz, Sezin Doğan Çakır, Hülya Kaynak, Emine Kartal Baykan, Mehmet Güven, Murat Çalapkulu, Onur Daloğlu, Yasemin Aydoğan Ünsal, Özlem Kanburoğlu, Selin Çakmak Demir, Dilek Gogas Yavuz, Zeynep Çetin, Ayşen Akkurt Kocaeli, Zeliha Hekimsoy, Mithat Mızrak, Zehra Candan, Erman Çakal, Gülce Ecem Kılıç, Göknur Yorulmaz, Mine Adaş, Zeliha Yarar, Pınar Akhanlı, Metin Güçlü, Muhammet Cüneyt Bilginer, Adnan Batman, Sevdenur Taşkın, Sema Hepşen, Aysen Akalın, Serpil Çiftel, Anna Abbasgholizadeh, Oğuzhan Deyneli, Zehra Erdemir, Gamze Gelir Çavdar, Yusuf Kayhan, Taner Bayraktaroğlu, Güven Özkaya

**Affiliations:** 1 Department of Endocrinology, Bursa Uludağ University Medical School, Bursa, Türkiye; 2 Department of Endocrinology, Kocaeli University Medical School, İzmit, Türkiye; 3 Department of Endocrinology, Dr. Ersin Arslan Training and Research Hospital, Şehitkamil, Türkiye; 4 Department of Endocrinology, Manisa Celal Bayar University Medical School, Manisa, Türkiye; 5 Department of Endocrinology, Fırat University Medical School, Elazığ, Türkiye; 6 Dr. Abdurrahman Yurtaslan Ankara Oncology Training and Research Hospital, Ankara, Türkiye; 7 Department of Endocrinology, Recep Tayyip Erdoğan University Training and Research Hospital, Rize, Türkiye; 8 Department of Endocrinology, Sinop Atatürk State Hospital, Sinop, Türkiye; 9 Department of Endocrinology, Ankara Etlik State Hospital, Ankara, Türkiye; 10 Department of Endocrinology, İzmir Ekonomi University Medicalpoint Hospital, İzmir, Türkiye; 11 Department of Endocrinology, Ankara Training and Research Hospital, Ankara, Türkiye; 12 Department of Endocrinology, Eskişehir Osmangazi University Medical School, Eskişehir, Türkiye; 13 Department of Endocrinology, İstanbul Prof. Dr. Cemil Taşçıoğlu City Hospital, İstanbul, Türkiye; 14 Department of Endocrinology, Konya Necmettin Erbakan Training and Research Hospital, Konya, Türkiye; 15 Department of Endocrinology, Erzurum City Hospital, Erzurum, Türkiye; 16 Department of Endocrinology, Diyarbakır Gazi Yaşargil Training and Research Hospital, Diyarbakır, Turkey; 17 Iğdır Dr. Nevruz, Erez State Hospital, Iğdır, Türkiye; 18 Department of Endocrinology, University of Health Sciences Bursa Yüksek İhtisas Training and Research Hospital, Bursa, Türkiye; 19 Department of Endocrinology, Ankara Yenimahalle Training and Research Hospital, Ankara, Türkiye; 20 Department of Endocrinology, Karadeniz Teknik University Medical School, Trabzon, Türkiye; 21 Department of Endocrinology, Koç University Hospital, İstanbul, Türkiye; 22 Department of Endocrinology, Marmara University Medical School, Istanbul, Turkey; 23 Department of Endocrinology, Amasya University Sabuncuoğlu Şerefeddin Training and Research Hospital, Amasya, Türkiye; 24 Department of Endocrinology, Bursa City Hospital, Bursa, Türkiye; 25 Department of Endocrinology, Ankara Etlik City Hospital, Ankara, Türkiye; 26 Department of Endocrinology, İzmir Bozyaka Training and Research Hospital, İzmir, Türkiye; 27 Department of Endocrinology, Ondokuz Mayıs University Medical School, Samsun, Türkiye; 28 Department of Endocrinology, Zonguldak Bülent, Ecevit University Medical School, Zonguldak, Türkiye

**Keywords:** Diabetes mellitus, Type 1 diabetes, Type 2 diabetes, Osteoporosis, Bone mineral density, FRAX

## Abstract

**Summary:**

We assessed bone health in over 2500 adults with diabetes across Türkiye. T1DM patients had lower BMD, while T2DM patients showed higher fracture risk despite higher BMD. Several modifiable factors were linked to osteoporosis. These findings support personalized bone assessments in diabetic populations.

**Background:**

Diabetes mellitus (DM) is a recognized risk factor for bone fragility. While type 1 DM (T1DM) is typically associated with low bone mineral density (BMD), type 2 DM (T2DM) often presents with higher BMD despite increased fracture risk. Large-scale comparative studies remain limited.

**Objective:**

To evaluate bone health, including osteopenia, osteoporosis, and fracture risk, in adults with T1DM and T2DM.

**Methods:**

This multicenter, cross-sectional study included 2562 patients (224 with T1DM, 2338 with T2DM) from 27 centers across Türkiye. BMD was assessed using dual-energy X-ray absorptiometry (DXA) and fracture risk was estimated via the Turkish-adapted FRAX® algorithm in patients aged ≥ 40 years. Multinomial logistic regression was used to identify independent predictors of low BMD.

**Results:**

Osteoporosis prevalence was 5.5% in T1DM and 9.6% in T2DM (femoral neck T-score). Adjusted BMD was significantly lower in T1DM at all skeletal sites, while FRAX-based fracture risk and fall-related fractures were higher in T2DM. Independent predictors of osteoporosis included older age, female sex, lower BMI, reduced calcium levels, corticosteroid use, albuminuria, hypertension, and less frequent sodium-glucose cotransporter-2 (SGLT2) inhibitor use. T1DM was independently associated with osteopenia but not osteoporosis.

**Conclusion:**

This multicenter study demonstrates distinct patterns of BMD and fracture risk across diabetes subtypes and supports individualized bone health assessment in routine diabetes care.

## Introduction

Osteoporosis and related fractures are major contributors to morbidity, disability, and healthcare burden worldwide [1, 2]. As global life expectancy increases, the incidence of osteoporotic fractures is projected to rise, posing significant challenges for health systems. Identifying high-risk populations and modifiable contributors to bone fragility has therefore become a public health priority [3].

Diabetes mellitus (DM), a chronic metabolic disorder with a rising global prevalence, has gained recognition as a risk factor for skeletal fragility. In Türkiye, adult diabetes prevalence is estimated at 15.9%, among the highest in Europe [4]. While the vascular complications of diabetes are well known, its skeletal consequences remain underappreciated. Both type 1 (T1DM) and type 2 diabetes mellitus (T2DM) are associated with increased fracture risk, but through distinct mechanisms [5–9].

In T1DM, insulin deficiency and impaired bone formation lead to reduced bone mineral density (BMD), whereas T2DM is often characterized by preserved or increased BMD despite a paradoxical increase in fracture risk [5, 8, 9]. This dissociation may reflect impaired bone quality due to chronic hyperglycemia, accumulation of advanced glycation end-products (AGEs), and increased fall risk secondary to diabetic complications such as peripheral neuropathy and retinopathy [10–12].

Most previous studies in Türkiye have been limited to small, single-center samples—frequently focused on postmenopausal women with T2DM—resulting in a lack of generalizable data [6]. Comparative national data on BMD and fracture risk across diabetes subtypes, and studies exploring modifiable risk factors in diabetic populations, remain scarce.

To address these gaps, we conducted a large, multicenter study across diverse regions of Türkiye to evaluate BMD and fracture risk in adults with T1DM and T2DM across Türkiye. Additionally, we aimed to identify clinical, biochemical, and treatment-related factors associated with low BMD and increased fracture risk, with the goal of informing individualized screening and management strategies in this high-risk population.

## Material and methods

### Study design and setting

This multicenter, retrospective, cross-sectional study was conducted between October 2023 and April 2024 across 27 endocrinology centers representing all major geographical regions of Türkiye. Centers were selected based on geographic representativeness, patient volume, and availability of dual-energy X-ray absorptiometry (DXA) equipment. Patients were identified through retrospective review of clinical and DXA records at each participating center. The primary aim was to assess bone health status and identify associated clinical and biochemical risk factors among adult patients with either T1DM or T2DM.

### Study population

**Fig. 1:**
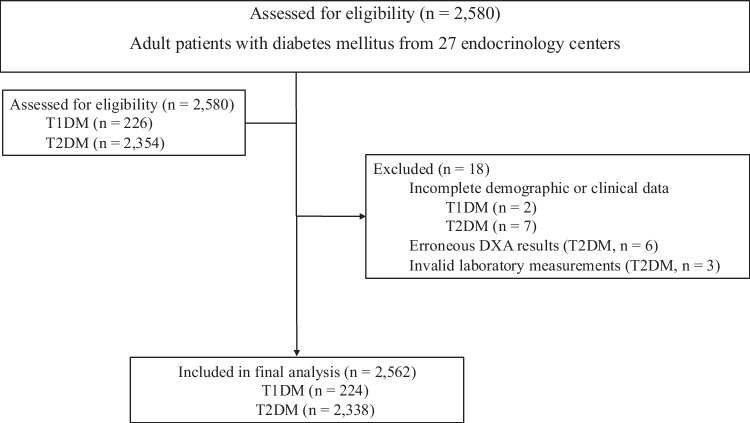
Study flow diagram of patient selection

A total of 2580 adult patients with diabetes mellitus were initially identified, including 226 with T1DM and 2354 with T2DM. T1DM and T2DM were distinguished based on standard clinical criteria. T1DM was defined by early age at onset, insulin dependence from diagnosis, and a clinical presentation consistent with autoimmune diabetes, supported by autoantibody or C-peptid testing when available. T2DM was defined by adult-onset hyperglycemia without features of autoimmune diabetes and typically without insulin requirement at diagnosis. After excluding two T1DM and sixteen T2DM patients due to missing or inconsistent data, the final analysis included 2562 participants. The patient selection process and reasons for exclusion are summarized in Fig. 1. As all eligible patients with available data were included, no a priori sample size calculation was performed.

Eligible individuals were aged ≥ 18 years and had a confirmed diagnosis of T1DM for at least two years or T2DM for a minimum of six months, based on standard clinical and biochemical criteria. Participants had to be under regular follow-up at the same healthcare center for at least six months and to have completed BMD assessment and standard laboratory testing within the preceding six months.

Patients were excluded if they lacked BMD or laboratory data, had diabetes types other than T1DM or T2DM, had active malignancy, or had undergone organ transplantation. A structured questionnaire was administered either by phone or in person to assess osteoporosis-related risk factors. Only individuals with complete clinical, laboratory, and DXA data were included in the final cohort. For each participant, data corresponded to a single clinical visit, consistent with the retrospective cross-sectional design. DXA scans were obtained based on clinical indications or physician judgement rather than routine screening and therefore reflect individuals for whom BMD assessment was considered clinically appropriate.

To ensure consistency, all centers used a standardized electronic data collection form. Participating sites were instructed to apply uniform units of measurement across all variables. Centralized quality checks were applied to exclude patients with incomplete or erroneous entries from the analysis.

### Data collection and measurement

Demographic and clinical data collected included age, sex, diabetes duration, age at menopause (if applicable), smoking status, and alcohol consumption. Pharmacologic information encompassed current antidiabetic medications—such as insulin, thiazolidinediones (TZDs), and sodium-glucose cotransporter-2 (SGLT2) inhibitors—as well as drugs known to affect bone metabolism, including proton pump inhibitors (PPIs), diuretics, and corticosteroids.

The presence of diabetic microvascular complications (retinopathy, nephropathy, and neuropathy) and macrovascular conditions (coronary artery disease, cerebrovascular disease, and peripheral arterial disease) was assessed based on clinical history and available medical documentation at each participating center.

Anthropometric measurements, laboratory values, and DXA results were extracted from clinical records; questionnaire data collected by phone or in person did not affect measured variables. Laboratory assessments included fasting plasma glucose (FPG), glycated hemoglobin (HbA1c), estimated glomerular filtration rate (eGFR), liver enzymes (Aspartate aminotransferase [AST] and alanine aminotransferase [ALT]), corrected serum calcium, phosphorus, magnesium, 25-hydroxyvitamin D (25 (OH) vitamin D), parathyroid hormone (PTH), thyroid-stimulating hormone (TSH), and the 24-h urinary calcium-to-creatinine ratio.

### BMD and fracture risk assessment

BMD was measured at the lumbar spine, femoral neck, and total hip using DXA scanners from a single manufacturer (Hologic®, Hologic Inc., USA) across all centers. Calibration procedures were performed in accordance with manufacturer guidelines and local quality control standards. No between-scanner cross-calibration was performed across centers.

T-scores were derived based on the NHANES III reference database for White women aged 20–29 years, as recommended by the International Osteoporosis Foundation (IOF) Working Group [13]. According to World Health Organization (WHO) criteria, osteopenia was defined as a T-score between –1.0 and –2.5, and osteoporosis as a T-score ≤ –2.5.

Fracture risk was estimated using the Turkish version of the Fracture Risk Assessment Tool (FRAX®) algorithm in patients aged ≥ 40 years, following guideline recommendations (with intervention thresholds defined in the FRAX-TURK study) [14]. The algorithm calculates the 10-year probability of major osteoporotic and hip fractures based on clinical risk factors, with or without BMD input. In patients diagnosed with osteoporosis, additional subgroup analyses were conducted to explore associations between BMD and clinical parameters such as HbA1c, diabetes duration, and the presence of microvascular or macrovascular complications.

### Statistical analysis

Statistical analyses were performed using IBM SPSS Statistics for Windows, Version 29.0.2.0 (IBM Corp., Armonk, NY). Descriptive statistics were expressed as mean ± standard deviation (SD) for continuous variables and as frequencies with percentages for categorical variables.

Comparisons between patients with T1DM and T2DM were conducted using independent samples t-tests for continuous variables and Pearson’s chi-square or Fisher’s exact test for categorical variables, as appropriate. For multiple comparisons among more than two groups, the Bonferroni correction was applied. Effect sizes were reported using Cohen’s d for continuous variables, eta squared for ANOVA, and Cramer’s V or Phi coefficient for categorical variables.

Analysis of covariance (ANCOVA) was used to compare BMD values between T1DM and T2DM groups after adjusting for age, sex, BMI, and diabetes duration. Adjusted means and standard errors (SE) were calculated using least squares estimation.

To evaluate factors associated with bone health status (normal BMD, osteopenia, osteoporosis), multinomial logistic regression analysis was performed. The results were presented as odds ratios (OR) with 95% confidence intervals (CI). A p-value of < 0.05 was considered statistically significant. For all regression analyses, BMD categories were defined using the minimum T-score across the lumbar spine, femoral neck, and total hip.

*Ethical considerations.*

The study was conducted following the principles of the Declaration of Helsinki. Ethical approval was obtained from all participating centers' local institutional review boards. Following the acquisition of site-specific approvals, final approval for the study protocol was granted by the Ethics Committee of Uludağ University Faculty of Medicine (Decision number: 2011-KAEK-26/452, Date: 6th July 2023), which served as the coordinating center. Written informed consent was waived owing to the retrospective nature of the study and use of anonymized records.

## Results

### Baseline characteristics of the study population

A total of 2562 patients were included in the analysis, comprising 224 individuals with T1DM and 2338 with T2DM. Patients with T2DM were significantly older (60.81 ± 10.1 vs. 37.44 ± 13.11 years, *p* < 0.001), had a higher BMI (31.02 ± 6.08 vs. 24.67 ± 4.5 kg/m^2^, *p* < 0.001), and a shorter duration of diabetes (141.17 ± 98.17 vs. 175.96 ± 124.45 months, *p* < 0.001) compared to those with T1DM.

The proportion of female participants was higher in the T2DM group (73.2% vs. 64.7%, *p* = 0.007). Smoking was more prevalent among T1DM patients, whereas hypertension (56.2% vs. 14.7%, *p* < 0.001) and fracture history were more common in T2DM (9.9% vs. 5.4%, p = 0.026). Macrovascular complications such as coronary artery disease and cerebrovascular disease were also more frequently observed in the T2DM group (*p* < 0.001 for both). While the prevalence of nephropathy was similar between groups, retinopathy and polyneuropathy were slightly more common among T2DM patients, though the differences were not statistically significant. Detailed demographic and clinical characteristics of the study population are summarized in Table 1.

**Table 1:** Demographic and Clinical Characteristics of the Study Population

Variable	T1DM (n = 224)	T2DM (n = 2,338)	Total (n = 2,562)	p-value	Effect size
Age, years	37.4 ± 13.1	60.8 ± 10.1	58.8 ± 12.3	< 0.001	−2.247
Female	145 (64.7%)	1,711 (73.2%)	1,855 (72.4%)	0.007	0.053
Menopause age, years (only in women)	46.8 ± 5.5	47.19 ± 5.2	47.18 ± 5.2	0.707	−0.072
Duration of diabetes, months	176.0 ± 124.4	141.2 ± 98.2	144.2 ± 101.2	< 0.001	0.345
Smoking Current smoker Ex smoker Non-smoker	54 (24.2%)	315 (13.5%)	369 (14.4%)	< 0.001	0.097
	23 (10.3%)	436 (18.7%)	459 (18.0%)		
	146 (65.5%)	1,582 (67.8%)	1,728 (67.6%)		
Alcohol use	3 (1.3%)	25 (1.1%)	28 (1.1%)	0.731	−0.007
BMI, kg/m^2^	24.7 ± 4.5	31.0 ± 6.1	30.5 ± 6.2	< 0.001	−1.065
Fracture history	12 (5.4%)	232 (9.9%)	244(9.5%)	0.026	0.044
Retinopathy	47 (21.1%)	412 (17.7%)	459 (18.0%)	0.205	0.025
Polyneuropathy	57 (25.6%)	737 (31.6%)	794 (31.0%)	0.064	0.037
Nephropathy	55 (24.8%)	574 (24.7%)	629 (24.7%)	0.972	−0.001
Hypertension	33 (14.7%)	1,312 (56.2%)	1,345 (52.6%)	< 0.001	0.235
Coronary artery disease	10 (4.5%)	497 (21.3%)	507 (19.8%)	< 0.001	0.119
Cerebrovascular disease	0 (0%)	79 (3.4%)	79 (3.1%)	0.005	0.055
Peripheral arterial disease	2 (0.9%)	40 (1.7%)	42 (1.6%)	0.357	0.018

T1DM, type 1 diabetes mellitus; T2DM, type 2 diabetes mellitus; BMI, body mass index

Cohen’s d (pooled standard deviation), Cramer’s V, and Phi effect sizes were presented

Data are presented as mean ± standard deviation or frequency (%)

### Comparative bone mineral density and fracture risk between diabetes types

According to WHO criteria based on femoral neck T-scores, osteoporosis was more frequently observed in patients with T2DM (9.6%) than in those with T1DM (5.5%) (*p* < 0.001). A similar trend was noted for lumbar spine osteoporosis (15.5% vs. 10.0%). However, when adjusted for age, sex, BMI, and diabetes duration, BMD values at all skeletal sites were significantly lower in patients with T1DM. Specifically, adjusted lumbar spine BMD was 1.03 ± 0.02 g/cm^2^ in T1DM and 1.08 ± 0.01 g/cm^2^ in T2DM (p = 0.002); adjusted total femur BMD was 0.88 ± 0.01 g/cm^2^ vs. 1.00 ± 0.01 g/cm^2^ (*p* < 0.001), and adjusted femoral neck BMD was 0.84 ± 0.01 g/cm^2^ vs. 0.91 ± 0.01 g/cm^2^ (*p* < 0.001).

Despite having higher adjusted BMD values, patients with T2DM exhibited significantly greater 10-year fracture risk as assessed by the FRAX® tool. Major osteoporotic fracture risk was 5.40 ± 4.43% in T2DM compared to 4.27 ± 2.44% in T1DM (*p* < 0.001), and hip fracture risk was 1.31 ± 2.98% vs. 0.66 ± 1.38%, respectively (*p* < 0.001). Moreover, a higher proportion of T2DM patients reported fall-related fractures (9.9%) compared to those with T1DM (5.4%, *p* = 0.026). All BMD and FRAX-based comparisons are detailed in Table 2.

**Table 2:** Comparison of Bone Mineral Density, Fracture Risk, and Osteoporosis/Osteopenia Prevalence Between Patients with Type 1 and Type 2 Diabetes Mellitus

	T1DM (n = 224)	T2DM (n = 2,338)	p-value	Effect size
Lumbar spine T-score	−0.79 ± 1.42	−0.73 ± 1.64	0.551	−0.037
Femoral neck T-score	−0.58 ± 1.14	−0.77 ± 1.38	0.028	0.134
Total femur T-score	−0.60 ± 1.06	−0.39 ± 1.28	0.006	−0.170
Lumbar spine BMD (g/cm^2^)	1.02 ± 0.18	1.05 ± 0.22	0.043	−0.122
Femoral neck BMD (g/cm^2^)	0.90 ± 0.16	0.88 ± 0.18	0.030	0.135
Total femur BMD (g/cm^2^)	0.91 ± 0.15	0.97 ± 0.18	< 0.001	−0.306
Adjusted lumbar spine BMD (g/cm^2^)*	1.03 ± 0.02	1.08 ± 0.01	0.002	0.004
Adjusted femoral neck BMD (g/cm^2^)*	0.84 ± 0.01	0.91 ± 0.01	< 0.001	0.009
Adjusted total femur BMD (g/cm^2^)*	0.88 ± 0.01	1.00 ± 0.01	< 0.001	0.030
FRAX major osteoporotic fracture risk (%)**	4.27 ± 2.44	5.40 ± 4.43	< 0.001	−0.263
FRAX hip fracture risk (%)**	0.66 ± 1.38	1.31 ± 2.98	< 0.001	−0.226
Lumbar spine				
*Normal BMD*	120 (54.3%)	1218 (52.8%)	0.071	0.046
*Osteopenia*	79 (35.7%)	730 (31.7%)		
*Osteoporosis*	22 (10.0%)	358 (15.5%)		
Femoral neck				
*Normal BMD*	142 (65.1%)	1191 (51.8%)	< 0.001***	0.077
*Osteopenia*	64 (29.4%)	886 (38.6%)		
*Osteoporosis*	12 (5.5%)	221 (9.6%)		
History of fall-related fractures	12 (5.4%)	232 (9.9%)	0.026	0.044

T1DM, type 1 diabetes mellitus; T2DM, type 2 diabetes mellitus; BMD, bone mineral density; FRAX, Fracture Risk Assessment Tool

Descriptive statistics are expressed as mean ± standard deviation or frequency with %. Where appropriate, statistical significance was assessed using independent samples t-tests or Pearson chi-square tests. Statistical significance was set at p < 0.05. Cohen’s d (pooled standard deviation), Partial eta squared, Cramer’s V, and Phi effect sizes were presented

^*^Adjusted BMD values are corrected for age, sex, BMI, and diabetes duration. Descriptive statistics are given as least square mean ± standard error

^**^ FRAX scores were calculated only for individuals aged ≥ 40 years; therefore, FRAX values for T1DM reflect the subgroup of patients aged 40 years and older (n = 96)

^***^According to pairwise comparisons with the Bonferroni-adjusted p-values, significant differences were found between the groups in the Normal BMD, Osteopenia, and Osteoporosis percentages

### Prevalence and determinants of low BMD

Among the total study population, 809 patients (31.6%) were classified as having osteopenia and 380 patients (14.8%) as having osteoporosis, based on lumbar spine T-scores **(**Table 3**)**. Increasing age was significantly associated with lower BMD status: patients in the osteoporosis group had a mean age of 62.89 ± 10.6 years, compared to 59.55 ± 12.2 years in the osteopenia group and 57.05 ± 12.5 years in those with normal BMD (*p* < 0.001). Similarly, female sex was increasingly prevalent across BMD categories, rising from 65.5% in the normal BMD group to 87.1% in the osteoporosis group (*p* < 0.001). BMI showed a strong inverse relationship with BMD status, decreasing progressively from 31.42 ± 6.17 kg/m^2^ in patients with normal BMD to 28.09 ± 5.69 kg/m^2^ in those with osteoporosis (*p* < 0.001). Interestingly, the duration of diabetes was slightly shorter in the osteoporosis group (128.7 ± 97.2 months) than in the normal BMD group (150.2 ± 101.2 months, *p* = 0.001), suggesting that factors beyond chronic hyperglycemia may contribute to bone loss in certain patients. Microalbuminuria and macroalbuminuria were also more prevalent in patients with osteoporosis compared to those with normal BMD (*p* = 0.023 and *p* = 0.030, respectively), indicating a potential link between diabetic microvascular complications and skeletal fragility.

**Table 3:** Comparison of Demographic, Clinical, and Laboratory Characteristics Among Patients with Normal BMD, Osteopenia, and Osteoporosis Based on Lumbar Spine Measurements

Variable	Normal BMD (n = 1,338)	Osteopenia (n = 809)	Osteoporosis (n = 380)	p-value	Effect size
Demographic Characteristics					
Age (years)	57.1 ± 12.5^a^	59.6 ± 12.2^b^	62.9 ± 10.6^c^	< 0.001	0.028
Female	877 (65.5%)^a^	624 (77.1%)^b^	331 (87.1%)^c^	< 0.001	0.180
Smoking				< 0.001	0.072
Current smoker	209 (15.6%)^a^	122 (15.1%)^a^	35 (9.3%)^b^		
Ex smoker	274 (20.5%)^a^	126 (15.6%)^b^	54 (14.3%)^b^		
Non-smoker	854 (63.9%)^a^	558 (69.2%)^b^	289 (76.5%)^c^		
BMI (kg/m^2^)	31.4 ± 6.2^a^	29.9 ± 6.1^b^	28.1 ± 5.7^c^	< 0.001	0.037
Duration of diabetes (months)	150.2 ± 101.2^a^	142.5 ± 102.4^ab^	128.7 ± 97.2^b^	0.001	0.006
Fracture history	92 (6.9%)^a^	70 (8.7%)^ab^	68 (17.9%)^b^	p < 0.001	0.132
Laboratory Characteristics					
A1C (%)	8.2 ± 2.1	8.0 ± 2.0	8.2 ± 2.2	0.100	0.002
Creatinin (mg/dL)	0.84 ± 0.35	0.83 ± 0.4	0.86 ± 0.49	0.547	0.001
Calcium (mg/dL)	9.6 ± 0.5^a^	9.6 ± 0.6^a^	9.4 ± 0.7^b^	< 0.001	0.011
Phosphorus (mg/dL)	3.6 ± 0.6	3.6 ± 0.7	3.5 ± 0.7	0.051	0.002
25(OH)Vitamin D (ng/mL)	20.8 ± 11.6	20.9 ± 12.1	21.1 ± 12.9	0.918	0.001
PTH (pg/mL)	50.6 ± 32.4^a^	55.4 ± 50.1^ab^	60.4 ± 50.4^b^	< 0.001	0.007
TSH (mIU/L)	2.1 ± 2.1	2.0 ± 1.7	1.9 ± 1.8	0.094	0.002
Total cholesterol (mg/dL)	191.6 ± 47.5	195.1 ± 46.6	192.8 ± 41	0.248	0.001
HDL cholesterol (mg/dL)	48.4 ± 12.9^a^	50.9 ± 14.7^b^	49.6 ± 14.4^ab^	< 0.001	0.006
LDL cholesterol (mg/dL)	113.3 ± 39.3	115.8 ± 41.1	112.8 ± 34.9	0.292	0.001
Triglyceride (mg/dL)	179.8 ± 133.3	174.9 ± 145.6	166.1 ± 105.7	0.205	0.001
Antidiabetic drugs					
TZD use	129 (9.6%)	63 (7.8%)	34 (8.9%)	0.351	0.029
SGLT2 inhibitor use	518 (38.7%)^a^	262 (32.4%)^b^	82 (21.6%)^c^	< 0.001	0.126
Insulin use	716 (53.6%)^a^	382 (47.5%)^b^	169 (44.6%)^b^	0.001	0.073
Diabetic complications					
Retinopathy	261 (19.6%)	125 (15.5%)	66 (17.5%)	0.055	0.048
Polyneuropathy	416 (31.2%)	232 (28.7%)	130 (34.2%)	0.149	0.039
Microalbuminuria	278 (20.9%)^ab^	141 (17.5%)^b^	91 (24.2%)^a^	0.023	0.055
Macroalbuminuria	70 (5.3%)^a^	44 (5.5%)^ab^	33 (8.8%)^b^	0.030	0.053
Coronary artery disease	273 (20.4%)	153 (18.9%)	80 (21.1%)	0.608	0.020
Cerebrovascular disease	36 (2.7%)	33 (4.1%)	8 (2.1%)	0.098	0.043
Peripheral arterial disease	21 (1.6%)	15 (1.9%)	5 (1.3%)	0.771	0.014
Hypertension	716 (53.6%)	418 (51.7%)	189 (49.9%)	0.385	0.028

BMD, bone mineral density; BMI, body mass index; A1c, hemoglobin A1c; PTH, parathyroid hormone; TSH, thyroid-stimulating hormone; HDL, high-density lipoprotein; LDL, low density lipoprotein; TZD, thiazolidinedione; SGLT2, sodium-glucose cotransporter-2

^abc^ The superscripts show the results of pairwise comparisons using the Bonferroni test between groups; values with unlike letters were significantly different among groups. Data are presented as mean ± standard deviation or frequency with percentage. Eta square, Cramer’s V, and Phi effect sizes were presented. Statistical significance was evaluated across all three groups using ANOVA or the Pearson chi-square test as appropriate

The distribution of medications affecting bone metabolism varied significantly across BMD categories (Table 4). The use of SGLT2 inhibitors was highest among patients with normal BMD (38.7%) and progressively declined in those with osteopenia (32.4%) and osteoporosis (21.6%) (*p* < 0.001). In contrast, PPI use was more frequent in patients with osteopenia (27.9%) compared to those with normal BMD (24.4%) and osteoporosis (23.2%) (*p* = 0.009). Corticosteroid use was relatively low overall but showed a non-significant trend toward higher prevalence in the osteoporosis group (4.5%) compared to the others (*p* = 0.173). Insulin and diuretic use were more common among patients with lower BMD, whereas TZD use did not differ significantly across groups.

**Table 4:** Drugs affecting bone metabolism and antidiabetic medications among patients with type 1 and type 2 diabetes mellitus

Medication	T1DM (n = 224)	T2DM (n = 2,338)	Total (n = 2,562)	p-value	Effect size
TZD	1 (0.4%)	230 (9.9%)	231 (9.0%)	< 0.001	0.093
SGLT2 inhibitors	2 (0.9%)	872 (37.3%)	874 (34.1%)	< 0.001	0.216
Insulin	224 (100%)	1,063 (45.6%)	1,287 (50.4%)	< 0.001	0.308
PPI	23 (10.3%)	627 (26.8%)	650 (25.4%)	< 0.001	0.107
Diuretics	10 (4.5%)	411 (17.6%)	421 (16.4%)	< 0.001	0.100
Corticosteroids	8 (3.6%)	97 (4.1%)	105 (4.1%)	0.677	0.008

T1DM, type 1 diabetes mellitus; T2DM, type 2 diabetes mellitus; TZD, thiazolidinedione; SGLT2, sodium-glucose cotransporter-2; PPI, proton pump inhibitor

Data are presented as frequency and percentage. Phi coefficent were presented as effect size

Biochemical parameters also varied according to BMD status, with several markers showing significant associations **(**Table 5**).** Serum calcium levels were lower in the osteoporosis group (9.40 ± 0.74 mg/dL) compared to patients with normal BMD (9.58 ± 0.52 mg/dL, *p* < 0.001), while phosphorus and creatinine levels did not differ significantly across groups. Although 25-hydroxyvitamin D levels did not show a significant overall difference among groups (*p* = 0.918), PTH levels were higher in the osteoporosis group (60.4 ± 50.4 pg/mL) than in the normal BMD group (50.6 ± 32.4 pg/mL, *p* < 0.001). TSH, lipid parameters, and eGFR were comparable across BMD categories.

**Table 5:** Comparison of biochemical variables in type 1 and type 2 diabetic patients

Variables	T1DM (n = 224)	T2DM (n = 2,338)	p-value	Effect size
Fasting plasma glucose (mg/dL)	177.1 ± 74.3	151.0 ± 62.4	< 0.001	0.411
Postprandial glucose (mg/dL)	240.1 ± 92.0	203.8 ± 74.8	< 0.001	0.476
HbA1c (%)	9.1 ± 2.2	8.0 ± 2.0	< 0.001	0.556
Urea (mg/dL)	27.6 ± 14.0	33.5 ± 16.1	< 0.001	−0.369
Creatinine (mg/dL)	0.8 ± 0.3	0.8 ± 0.4	0.010	−0.181
eGFR (mL/min/1.73 m^2^)	104.3 ± 22.1	85.2 ± 22.6	< 0.001	0.848
Total cholesterol (mg/dL)	182.0 ± 45.9	194.0 ± 46.2	< 0.001	−0.258
LDL cholesterol (mg/dL)	106.3 ± 37.1	114.8 ± 39.4	0.001	−0.218
HDL cholesterol (mg/dL)	54.7 ± 16.5	48.9 ± 13.3	< 0.001	0.427
Triglycerides (mg/dL)	126.5 ± 75.6	180.9 ± 137.0	< 0.001	−0.409
Calcium (mg/dL)	9.3 ± 0.6	9.6 ± 0.6	< 0.001	−0.544
Phosphorus (mg/dL)	3.5 ± 0.8	3.6 ± 0.7	< 0.001	−0.278
Magnesium (mg/dL)	1.9 ± 0.2	1.9 ± 0.3	0.004	−0.166
25 (OH) Vitamin D (ng/mL)	18.5 ± 10.1	21.1 ± 12.2	0.002	−0.221
PTH (pg/mL)	59.7 ± 61.9	53.1 ± 39.4	0.148	0.157
TSH (µIU/mL)	2.2 ± 1.9	2.0 ± 2.0	0.148	0.102
Urinary calcium (mg/day)	85.6 ± 77.4	174.9 ± 138.5	< 0.001	−0.714

T1DM, type 1 diabetes mellitus; T2DM, type 2 diabetes mellitus; HbA1c, glycated hemoglobin; eGFR, estimated glomerular filtration rate; LDL, low density lipoprotein; HDL, high-density lipoprotein; PTH, parathyroid hormone; TSH, thyroid-stimulating hormone

Data are presented as mean ± standard deviation. Statistical significance is based on an independent samples t-test. Cohen’s d (pooled standard deviation) was given as an effect size

### Multivariate analysis of factors associated with bone health

To evaluate the independent associations of these variables with low BMD, a multinomial logistic regression analysis was performed. Variables found to be significant in univariate comparisons—including age, sex, BMI, diabetes duration, microvascular complications, medication use, and select biochemical markers—were included in the multivariate model. The results are presented in the following section.

Multinomial logistic regression analysis identified several independent predictors of osteopenia and osteoporosis, using normal BMD as the reference category (Table 6). Increasing age and female sex were strongly associated with a higher likelihood of both conditions. Each additional year of age increased the odds of osteopenia by 3.0% (OR = 1.030, 95% CI: 1.016–1.043) and osteoporosis by 4.6% (OR = 1.046, 95% CI: 1.028–1.064), while female sex was associated with more than twice the odds of osteopenia (OR = 2.119, 95% CI: 1.584–2.834; *p* < 0.001) and nearly five times the odds of osteoporosis (OR = 4.708, 95% CI: 3.038–7.296; *p* < 0.001).

**Table 6:** Multinomial logistic regression of Osteopenia, Osteoporosis, and Normal BMD

	Osteopenia vs Normal BMD		Osteoporosis vs Normal BMD	
	OR (95%CI)	p	OR (95%CI)	p
Age (years)	1.030(1.016–1.043)	< 0.001	1.046(1.028–1.064)	< 0.001
BMI (kg/m2)	0.938(0.920–0.957)	< 0.001	0.884(0.86–0.909)	< 0.001
Duration of diabetes, months (per SD increase)	0.836(0.738–0.946)	0.005	0.744(0.628–0.881)	0.001
T1DM vs T2DM	1.808(1.108–2.95)	0.018	0.717(0.347–1.478)	0.367
Gender (Female)	2.119(1.584–2.834)	< 0.001	4.708(3.038–7.296)	< 0.001
Fracture history	1.404 (0.947–2.082)	0.092	2.93 (1.88–4.565)	< 0.001
Current smoker vs non-smoker	1.157(0.846–1.583)	0.361	0.803(0.495–1.302)	0.373
Ex-smoker vs non-smoker	0.839(0.617–1.141)	0.263	0.725(0.47–1.118)	0.145
HbA1c (%)	0.975(0.917–1.036)	0.414	1.061(0.981–1.147)	0.139
Calcium (mg/dL)	1.034(0.86–1.244)	0.722	0.745(0.588–0.945)	0.015
Phosphorus (mg/dL)	1.050(0.888–1.241)	0.569	0.815(0.651–1.02)	0.074
PTH (pg/mL)	1.003(1.000–1.005)	0.063	1.003(0.999–1.006)	0.099
TSH (µIU/mL)	0.981(0.928–1.038)	0.507	0.947(0.87–1.031)	0.209
25 (OH) Vitamin D (ng/mL)	0.990(0.981–0.999)	0.028	0.994(0.982–1.005)	0.289
Creatinine (mg/dL)	1.167(0.74–1.84)	0.507	1.117(0.651–1.918)	0.688
eGFR (mL/min/1.73 m^2^)	1.001(0.992–1.009)	0.840	0.997(0.986–1.008)	0.536
HDL cholesterol (mg/dL)	1.005(0.997–1.013)	0.255	0.998(0.987–1.008)	0.675
SGLT2i	0.899(0.713–1.133)	0.367	0.511(0.364–0.719)	< 0.001
Insulin	0.806(0.62–1.048)	0.107	0.799(0.561–1.139)	0.215
TZD	0.891(0.611–1.297)	0.546	0.9(0.522–1.554)	0.706
Corticosteroids	1.187(0.682–2.065)	0.544	1.973(1.07–3.638)	0.029
Diuretics	0.802(0.591–1.088)	0.157	0.872(0.577–1.32)	0.518
PPI	1.398(1.087–1.797)	0.009	1.097(0.78–1.542)	0.596
Nephropathy	1.015(0.759–1.356)	0.920	1.277(0.871–1.873)	0.210
Retinopathy	0.877(0.636–1.209)	0.422	0.913(0.593–1.404)	0.678
Coronary artery disease	1.023(0.762–1.374)	0.879	1.247(0.843–1.846)	0.269
Cerebrovascular disease	1.396(0.755–2.581)	0.287	0.577(0.215–1.547)	0.274
Peripheral arterial disease	2.203(0.976–4.976)	0.057	1.505(0.446–5.072)	0.510
Hypertension	0.949(0.743–1.212)	0.674	0.704(0.506–0.979)	0.037

OR, Odds ratio; CI, Confidence interval; BMD, bone mineral density; BMI, body mass index; HbA1c, glycated hemoglobin; PTH, parathyroid hormone; TSH, thyroid-stimulating hormone; eGFR, estimated glomerular filtration rate; HDL, high-density lipoprotein; T1DM, type 1 diabetes mellitus; T2DM, type 2 diabetes mellitus; SGLT2i, sodium-glucose cotransporter-2 inhibitor; TZD, thiazolidinedione; PPI, proton pump inhibitor. Regression outcomes are based on BMD categories defined by the minimum T-score. Statistically significant values are shown in bold

BMI was a strong protective factor; each one-unit increase in BMI reduced the risk of osteopenia by 6.2% (OR = 0.938, *p* < 0.001) and osteoporosis by 11.6% (OR = 0.884, *p* < 0.001). SGLT2 inhibitor use was associated with a significantly lower risk of osteoporosis (OR = 0.511, *p* < 0.001), while PPI use increased the odds of osteopenia (OR = 1.398, *p* = 0.009). Corticosteroid use was also independently associated with osteoporosis (OR = 1.973, *p* = 0.029).

Biochemical parameters showed differential effects: higher serum calcium levels were protective against osteoporosis (OR = 0.745, *p* = 0.015), while 25-hydroxyvitamin D levels were modestly protective against osteopenia (OR = 0.990, *p* = 0.028). Interestingly, the presence of T1DM was associated with an increased risk of osteopenia (OR = 1.808, *p* = 0.018), and a history of fracture was strongly associated with osteoporosis (OR = 2.930, *p* < 0.001), whereas no significant association was observed with osteopenia, a pattern consistent with the greater clinical relevance of fracture history in established osteoporosis.

## Discussion

In this nationwide, multicenter study conducted across diverse regions of Türkiye, we evaluated BMD and fracture risk in adults with type 1 and type 2 diabetes mellitus. Osteoporosis was more frequently identified in individuals with T2DM based on unadjusted T-scores; however, patients with T1DM exhibited significantly lower adjusted BMD values across all skeletal sites. Conversely, fracture risk as estimated by the FRAX algorithm was higher among T2DM patients; however, it is important to note that FRAX does not fully capture diabetes-related fracture risk and may underestimate fracture probability in individuals with diabetes. In addition, several demographics, clinical, pharmacologic, and biochemical variables were independently associated with low BMD status. These findings underscore the multifactorial nature of diabetic bone disease and offer a nuanced perspective on osteoporosis risk stratification in individuals with diabetes.

The observed pattern of lower adjusted BMD in T1DM patients despite a higher prevalence of osteoporosis based on raw T-scores in T2DM is consistent with previous studies showing impaired bone formation and reduced peak bone mass in individuals with T1DM due to insulin deficiency and earlier disease onset [5, 15, 16]. In T2DM, the higher fracture rates are largely attributable to the older age of this population, rather than representing a true discordance between BMD and fracture risk. Nevertheless, reduced bone quality, increased fall risk, and the accumulation of AGEs described in the literature may further contribute to fracture susceptibility in T2DM patients [10–12]. Given that FRAX does not fully incorporate diabetes-related skeletal risk and tends to underestimate fracture probability, fracture risk assessment should therefore rely on clinical judgment integrating BMD findings together with diabetes-specific risk factors, rather than FRAX alone.

Several well-established demographic factors were found to be independently associated with low BMD in our cohort. Age and female sex were strongly predictive of both osteopenia and osteoporosis, consistent with previous findings that advancing age and postmenopausal status are key contributors to bone loss, regardless of diabetes status [17]. The protective effect of higher BMI, observed in both univariate and multivariate analyses, also aligns with prior literature suggesting that increased mechanical loading and estrogen production from adipose tissue may attenuate bone loss in overweight individuals [8]. However, it is important to note that while higher BMI may be associated with increased BMD, it does not necessarily confer protection against fracture, particularly in T2DM patients where bone quality is often compromised [18]. Interestingly, the prevalence of smoking was lower among patients with osteoporosis in our cohort, which contrasts with existing literature linking tobacco use to increased bone resorption and impaired bone quality [19]. This unexpected finding may reflect underreporting, survivor bias, or population-specific lifestyle factors, and highlights the need for further research into smoking-related bone outcomes in diabetic populations. Interestingly, shorter diabetes duration was observed in patients with osteoporosis, a finding that contrasts with the conventional view that longer disease exposure leads to greater skeletal deterioration. This may reflect the influence of non-glycemic factors such as age, nutritional status, and hormonal changes, particularly in older individuals with recent-onset diabetes.

Several commonly prescribed medications showed significant associations with BMD status in our cohort. The use of SGLT2 inhibitors was independently associated with a lower risk of osteoporosis in our cohort. While early clinical trials raised concerns—particularly regarding canagliflozin—more recent data suggest that the overall fracture risk associated with this drug class may not be increased and may vary depending on the agent and patient characteristics [20–22]. Our findings support the growing consensus that SGLT2 inhibitors do not exert a class-wide adverse effect on bone health and may be neutral or beneficial in real-world settings. In contrast, PPI use was associated with a higher prevalence of osteopenia, consistent with prior studies linking long-term PPI use to impaired calcium absorption and altered bone turnover [22]. Although the overall fracture risk associated with PPI use remains a subject of debate, our findings suggest that even subclinical changes in bone density may occur in patients receiving PPIs. Additionally, corticosteroid use, a well-known contributor to secondary osteoporosis, was significantly associated with osteoporosis in our study. This aligns with the robust evidence base showing that glucocorticoids promote bone resorption and inhibit bone formation, particularly when used chronically. These results underscore the importance of reviewing pharmacologic histories in patients with diabetes as part of comprehensive osteoporosis risk assessment strategies.

Both microvascular and macrovascular complications showed associations with bone mineral density in our cohort. The presence of microalbuminuria and macroalbuminuria was significantly more common among patients with osteoporosis compared to those with normal BMD, suggesting a potential link between diabetic microvascular complications and bone health. Although these associations did not remain significant in the multivariate analysis, the observed trend is consistent with emerging evidence that vascular health may play a critical role in skeletal integrity. Impaired bone perfusion, endothelial dysfunction, and chronic low-grade inflammation in diabetic microangiopathy have all been proposed as mechanisms contributing to reduced bone turnover and compromised bone quality [23]. Retinopathy and peripheral neuropathy, two other hallmark microvascular complications, were not extensively analyzed in our study due to limited data. However, prior literature has linked both conditions to increased fracture risk, largely through mechanisms such as impaired postural control, reduced physical activity, and shared metabolic derangements [23, 24]. Their presence may also reflect more advanced microvascular damage, which can indirectly affect bone quality. Similarly, macrovascular complications, including ischemic heart disease and stroke—were more prevalent among patients with low BMD. These conditions may reflect a broader systemic vascular dysfunction that compromises bone remodeling and integrity. Hypertension, also more frequently observed in the osteoporosis group, may contribute to bone fragility through increased arterial stiffness and disrupted microcirculation [23, 24]. While causal mechanisms remain speculative, these findings support the concept of a shared vascular–skeletal axis in diabetes.

Biochemical markers of bone metabolism also demonstrated differential associations with BMD status in our study. Lower serum calcium levels were significantly associated with osteoporosis in both univariate and multivariate analyses, underscoring the importance of calcium homeostasis in maintaining skeletal integrity. This finding aligns with previous evidence suggesting that even modest reductions in serum calcium may signal impaired bone turnover or secondary hyperparathyroidism [25]. Although mean serum 25-hydroxyvitamin D levels did not differ significantly between BMD groups, lower levels were independently associated with osteopenia in multivariate analysis. This finding may reflect the high background prevalence of vitamin D insufficiency in the general population, which can obscure between-group differences but still exert measurable skeletal effects [25]. PTH levels were higher in the osteoporosis group, consistent with a compensatory response to suboptimal calcium or vitamin D status, although they did not emerge as independent predictors in the regression model. These results suggest that subtle disturbances in mineral metabolism may contribute to early bone loss, particularly in patients with diabetes, and highlight the clinical value of routine screening for calcium and vitamin D status.

An unexpected finding in our study was that T1DM was independently associated with osteopenia; however, this association should be interpreted with caution, as the confidence intervals for osteopenia and osteoporosis substantially overlap. This is somewhat counterintuitive, as T1DM has been historically linked to greater skeletal fragility, lower peak bone mass, and reduced bone formation due to insulin deficiency and early disease onset [14, 15]. One possible explanation is that patients with T1DM in our cohort may have been younger, leaner, or otherwise protected against progressing to advanced bone loss, despite lower adjusted BMD values. Alternatively, the relatively smaller sample size of T1DM cases compared to T2DM may have limited the statistical power to detect associations with osteoporosis. This observation may also suggest that in certain populations, T1DM contributes more to early bone changes—reflected as osteopenia—rather than manifest osteoporosis. Further longitudinal studies are needed to explore the trajectory of bone loss in T1DM and to determine whether osteopenia in this group is a precursor to more severe skeletal outcomes.

This study has several limitations that should be acknowledged. First, its cross-sectional design precludes the establishment of temporal or causal relationships between clinical variables and BMD status. Second, the use of retrospective registry data may introduce selection bias and limit the availability of certain covariates, such as comprehensive records on diabetic retinopathy and neuropathy. Third, fracture risk was estimated using the FRAX algorithm, as direct data on incident fractures were not available. Fourth, the number of patients with T1DM was relatively small, which may limit the generalizability of findings specific to this subgroup. Fifth, because DXA testing was clinically indicated rather than systematically performed, selection bias is possible, and the findings may not generalize to all individuals with diabetes. Finally, medication-related associations, particularly those involving SGLT2 inhibitors and proton pump inhibitors, should be interpreted cautiously, as confounding by indication and unmeasured comorbidities may have influenced these findings. Overall, the findings should therefore be interpreted as associative and hypothesis-generating.

Despite these limitations, the study has notable strengths. It represents one of the largest multicenter analyses of bone health in diabetic patients in Türkiye, incorporating both T1DM and T2DM populations. Compared to previous studies conducted in Türkiye, which were often limited to postmenopausal women with T2DM and small, single-center samples [6], our multicenter design and inclusion of both sexes and diabetes types enhance the generalizability of the results to the broader diabetic population. This represents the most comprehensive dataset on diabetic bone health in Türkiye to date. The multicenter design, consistent use of Hologic DXA systems, and the integration of both BMD and FRAX-based risk assessment enhance the reliability and applicability of the findings. Furthermore, the simultaneous evaluation of demographic, pharmacologic, and biochemical predictors provides a comprehensive perspective on osteoporosis risk in this high-risk population.

In conclusion, this large multicenter study conducted across Türkiye offers a comprehensive assessment of bone health in adults with both type 1 and type 2 diabetes mellitus. Our findings emphasize the multifactorial nature of diabetic bone disease and highlight the need for individualized screening strategies that incorporate both BMD and clinical risk factors. By including a broad diabetic population across Türkiye, this study expands upon previous local research and provides real-world evidence to inform future prevention efforts.

## Data Availability

Data used in this study can be provided on reasonable request.
